# Identification of immune related gene signature for predicting prognosis of cholangiocarcinoma patients

**DOI:** 10.3389/fimmu.2023.1028404

**Published:** 2023-02-02

**Authors:** Zi-jian Zhang, Yun-peng Huang, Zhong-tao Liu, Yong-xiang Wang, Hui Zhou, Ke-xiong Hou, Ji-wang Tang, Li Xiong, Yu Wen, Sheng-fu Huang

**Affiliations:** Department of General Surgery, Second Xiangya Hospital, Central South University, Changsha, Hunan, China

**Keywords:** cholangiocarcinoma, immune, molecular subtype, RiskScore, immunotherapy

## Abstract

**Objective:**

To identify the gene subtypes related to immune cells of cholangiocarcinoma and construct an immune score model to predict the immunotherapy efficacy and prognosis for cholangiocarcinoma.

**Methods:**

Based on principal component analysis (PCA) algorithm, The Cancer Genome Atlas (TCGA)-cholangiocarcinoma, GSE107943 and E-MTAB-6389 datasets were combined as Joint data. Immune genes were downloaded from ImmPort. Univariate Cox survival analysis filtered prognostically associated immune genes, which would identify immune-related subtypes of cholangiocarcinoma. Least absolute shrinkage and selection operator (LASSO) further screened immune genes with prognosis values, and tumor immune score was calculated for patients with cholangiocarcinoma after the combination of the three datasets. Kaplan-Meier curve analysis determined the optimal cut-off value, which was applied for dividing cholangiocarcinoma patients into low and high immune score group. To explore the differences in tumor microenvironment and immunotherapy between immune cell-related subtypes and immune score groups of cholangiocarcinoma.

**Results:**

34 prognostic immune genes and three immunocell-related subtypes with statistically significant prognosis (IC1, IC2 and IC3) were identified. Among them, IC1 and IC3 showed higher immune cell infiltration, and IC3 may be more suitable for immunotherapy and chemotherapy. 10 immune genes with prognostic significance were screened by LASSO regression analysis, and a tumor immune score model was constructed. Kaplan-Meier (KM) and receiver operating characteristic (ROC) analysis showed that RiskScore had excellent prognostic prediction ability. Immunohistochemical analysis showed that 6 gene (NLRX1, AKT1, CSRP1, LEP, MUC4 and SEMA4B) of 10 genes were abnormal expressions between cancer and paracancer tissue. Immune cells infiltration in high immune score group was generally increased, and it was more suitable for chemotherapy. In GSE112366-Crohn’s disease dataset, 6 of 10 immune genes had expression differences between Crohn’s disease and healthy control. The area under ROC obtained 0.671 based on 10-immune gene signature. Moreover, the model had a sound performance in Crohn’s disease.

**Conclusion:**

The prediction of tumor immune score model in predicting immune microenvironment, immunotherapy and chemotherapy in patients with cholangiocarcinoma has shown its potential for indicating the effect of immunotherapy on patients with cholangiocarcinoma.

## 1 Introduction

Cholangiocarcinoma is a highly heterogeneous biliary malignancy originating from bile duct epithelial cells and can occur almost anywhere in the biliary tree. According to the anatomical location, it can be divided into intrahepatic cholan⁃giocarcinoma and perihilar cholangiocarcinoma (PCCA or Klatskin tumor) and distal cholangiocarcinoma, the incidence rates are 10%-20%, 50%-60% and 20%-30%, respectively (1–3). Cholangiocarcinoma is the second most common primary liver malignancy after hepatocellular carcinoma, accounting for about 15% of all primary liver tumors (4, 5). At the same time, cholangiocarcinoma is not sensitive to radiotherapy or chemotherapy, and there is a lack of effective targeting drugs, which is mainly due to a lack of understanding of the pathogenesis and heterogeneity of cholangiocarcinoma. Therefore, for cholangiocarcinoma tumors with high heterogeneity, it is the key to improve the clinical efficacy to find the biological characteristics common among tumors and different from normal hepatobiliary cells.

Cholangiocarcinoma is a proliferative tumor in connective tissue. Its tumor immune microenvironment contains immunosuppressive innate immune cells such as tumor-associated macrophages and myeloid suppressor cells and malignant tumor-associated fibroblasts, among which NK cells are usually less abundant (6). To avoid severe inflammatory responses due to continued exposure to gut microbiota and other antigens from the digestive system, the liver is in a state of chronic immune tolerance, which is mediated in part by Kupffer macrophages (7). Unique features of the microenvironment of cholangiocarcinoma may affect its responsiveness to immunotherapy, including the induction of fibroplasia, thereby limiting the penetration of drugs or immune cells. Tumor-related macrophages, other immune tolerance factors, dendritic cells express programmed cell death−ligand (PD−L1), which may also be used by tumor cells to up-regulate immune checkpoints. PD −1, T-cell immunoglobulin and mucin domain 3 (TIM3), Choline transporter-like protein 4 (CTL4), indoleamine 2,3-dioxygenase (IDO−1), Lymphocyte-activation gene 3 (LAG−3), etc., further leading to T cell depletion and promoting immune suppression and the progression of cholangiocarcinoma (8, 9). Screening candidate patients who could potentially benefit from receiving combination therapy and ICIs is highly necessary because taking ICIs is toxic and would pose economic burden (10). However, in what ways cholangiocarcinoma’s immune-related characteristics can be used to meet the immunotherapy demand of patients are not clear.

In this study, immune cell-related subtypes of cholangiocarcinoma were identified and an immune scoring model was constructed to analyze the differences in tumor microenvironment and immunotherapy among different cholangiocarcinoma immune cell-related subtypes and immunoevaluation groups. It is expected that the immune score model could predict the sensitivity of patients with cholangiocarcinoma to immunotherapy, and provide a new idea for the individualized treatment of patients with cholangiocarcinoma.

## 2 Materials and methods

### 2.1 Raw data

The RNA-seq data and the corresponding clinical follow-up information of cholangiocarcinoma were collected from The Cancer Genome Atlas (TCGA) database, Gene Expression Omnibus (GEO) database (GSE107943) and E-MTAB-6389 dataset (https://www.ebi.ac.uk/arrayexpress/experiments/EMTAB6389/). From the ImmPort a number of immune-related genes were downloaded (11).

Processing of the RNA-seq data was conducted following these criteria: 1) Removing the samples that did not have information on follow-up; 2) Converting the ENSEMBL IDs into gene symbols; and 3) The expression of multiple Gene symbols was taken as the median value.

The three datasets were combined to a dataset and named Joint data. And the sample information obtained after preprocessing of the three datasets was shown in **Table 1**.

**Table 1:** Sample information of three datasets.

Clinical Features	TCGA-CHOL	E- MTAB -6389	GSE107943
OS			
0	18	32	13
1	18	72	17
T Stage			
T1	19		
T2	12		
T3	5		
N Stage			
N0	26		
N1	5		
NX	5		
M Stage			
M0	28		
M1	5		
MX	3		
Stage			
I	19		
II	9		
III	1		
IV	7		
Age			
≤65	17		16
>65	19		14
Gender			
Male	16	49	24
Female	20	55	6

### 2.2 Univariate Cox survival regression analysis

The CoxPH function in R package was used to identify genes associated with prognosis (P<0.01) by univariate Cox analysis.

### 2.3 Consensus Cluster Plus

Using the Consensus Cluster Plus 1.52.0 in R package, we separately performed molecular subtyping for Joint data based prognosis-related immune genes (12). To complete 500 bootstraps, “pam” arithmetic and “pearson” distance were introduced here, with specimens (≥80%) of Joint data in each bootstrap. We set Cluster number k between 2 and 10, and the optimum k was identified as per cumulative distribution function (CDF) and CDF Delta area. Among the molecular subtypes, differences in their survival curves (KM curves) were examined.

### 2.4 Microenvironment cell populations-counter (MCP-Counter)

Amount of two stromal populations (endothelial cells and fibroblasts), eight immune populations (myeloid dendritic cells, CD8+ T cells, cytotoxic lymphocytes, natural killer cells, T cells, neutrophils, monocytic lineage, B lineages), immune-infiltrating cells was analyzed using the MCP-counter in R package in each sample (13).

### 2.5 Gene sets enrichment analysis (GSEA)

Using GSEA strategy in R package, 28 subpopulations of TILs were studied, including the main kinds associated with adaptive immunity: Th17 cells, follicular helper T cells (Tfh), central memory (Tcm), activated T cells, Th2 cells, immature, activated, and memory B cells, gamma delta T (Tgd) cells, T helper 1 (Th1) cells, effector memory (Tem) CD4+ and CD8+ T cells, regulatory T cells (Treg), and innate immunity-associated cell types like mast cells, MDSCs, macrophages, natural killer T (NKT) cells, activated, plasmacytoid, and immature dendritic cells (DCs),neutrophils, eosinophils, monocytes, NK cells.

### 2.6 ESTIMATE for Stromal and Immune cells in malignant tumors

R package ESTIMATE (14) computed the combination (ESTIMATE Score) of sufferers, the immunocyte infiltration (Immune Score), overall stroma level (Stromal Score) in the Joint data using Wilcox.test analysis to determine difference.

### 2.7 Developing a prognostic model for cholangiocarcinoma

Based on immune genes associated with prognosis, using the glmnet package, here LASSO regression was executed (15). To study cholangiocarcinoma samples’ prognosis, a formula was built as followed:


$$RiskScore{=}_{\sum }^{n}\beta i\times Expi$$

*Expi* means the expression of the *i* gene, β*i* means Cox regression coefficient of the *i* gene. Samples in Joint data were classed to low risk group (low group) and high risk group (high group) with the median of RiskScore. KM survival curve and ROC evaluated prognosis prediction for cholangiocarcinoma. Also, GSE112366 dataset was used for validation.

### 2.8 Tumor Immune Dysfunction and Exclusion (TIDE)

TIDE (16, 17) algorithm (http://tide.dfci.harvard.edu) was used to evaluate three cell types that limit T-cell invasion into tumors, including IFNG, myeloid suppressor cells (MDSC), and M2 subtypes of tumor-associated macrophages (TAM.M2), as well as exclusion of CTL by immunosuppressive factors (Exclusion), dysfunction of tumor infiltration cytotoxic T lymphocytes (CTL) (Dysfunction).

### 2.9 Drug sensitivity analysis

pRRophetic (18) was used to predict the sensitivity of 6 drugs to IC50. Sangerbox provided analytical assistance in this article (19).

### 2.10 Immunohistochemistry

Cholangiocarcinoma and paracancer specimens were desensitized in xylene and rehydrated in a series of graded alcohols. Endogenous peroxidase was blocked with 3% H2O2. The antigen was extracted after being heated in citric acid buffer. Sections were incubated overnight with primary antibodies anti-NLRX1 (ab107611, Abcam, Cambridge, MA, USA), anti-AKT1 (ab81283), anti-CSRP1 (ab175319), anti-LEP(ab16227), anti-MUC4 (ab307546) and anti-SEMA4B (ab118458, diluted 1:50) at 4°C. Horseradish peroxidase coupled secondary antibody was added and incubated at room temperature for 30min. Color development was performed with 3,3 ‘-diaminobenzidine (DAB) solution (Dako, Glostrup, Denmark).

## 3 Results

### 3.1 Identification of molecular subtype

**Figure 1:**
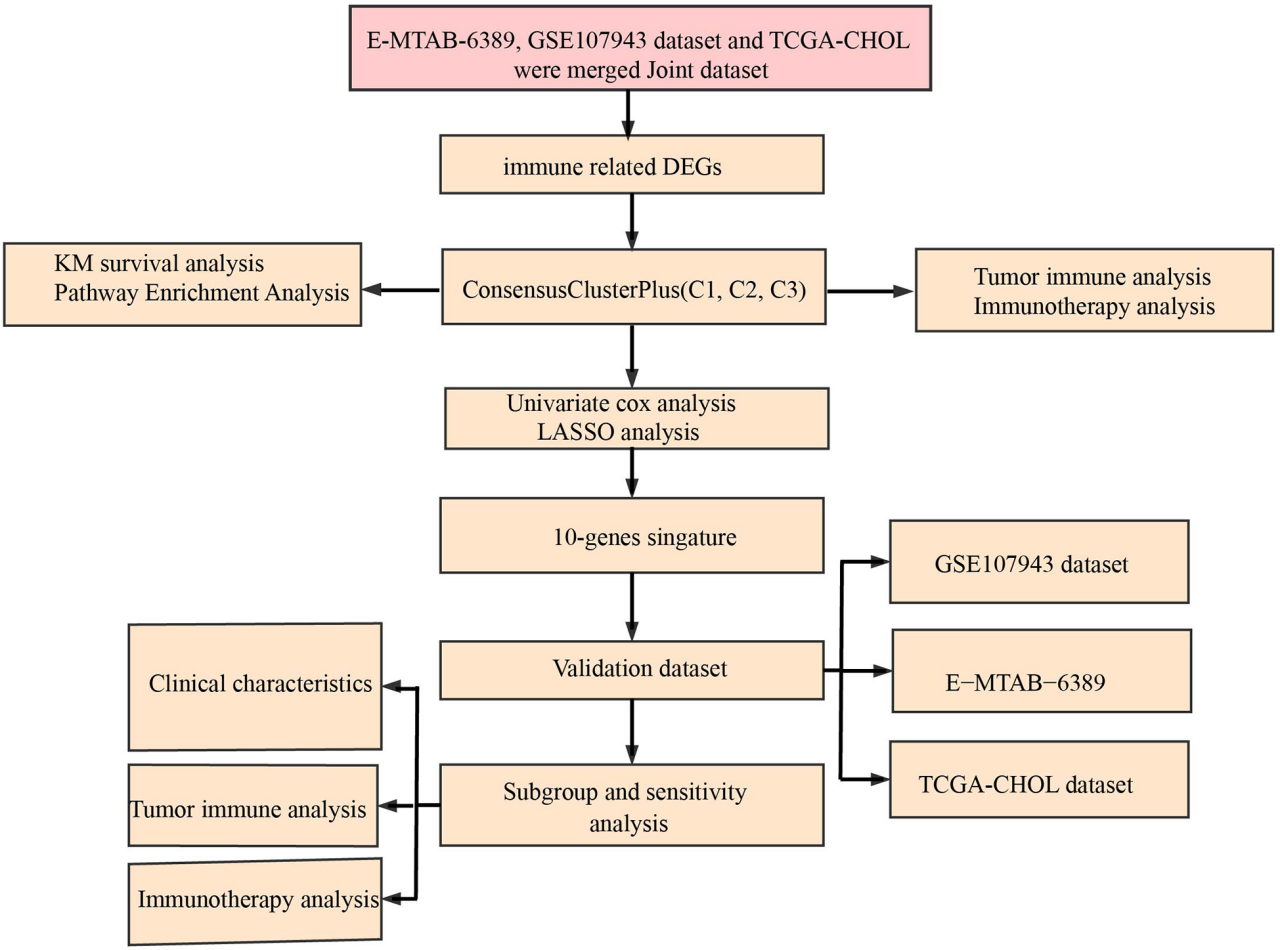
Working flow chart.

**Figure 2:**
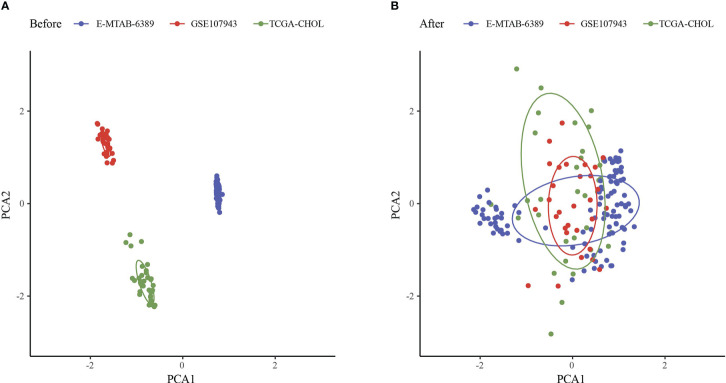
Principal component analysis. **(A)** PCA analysis before removal of batch effects. **(B)** PCA analysis after removal of batch effects.

**Figure 3:**
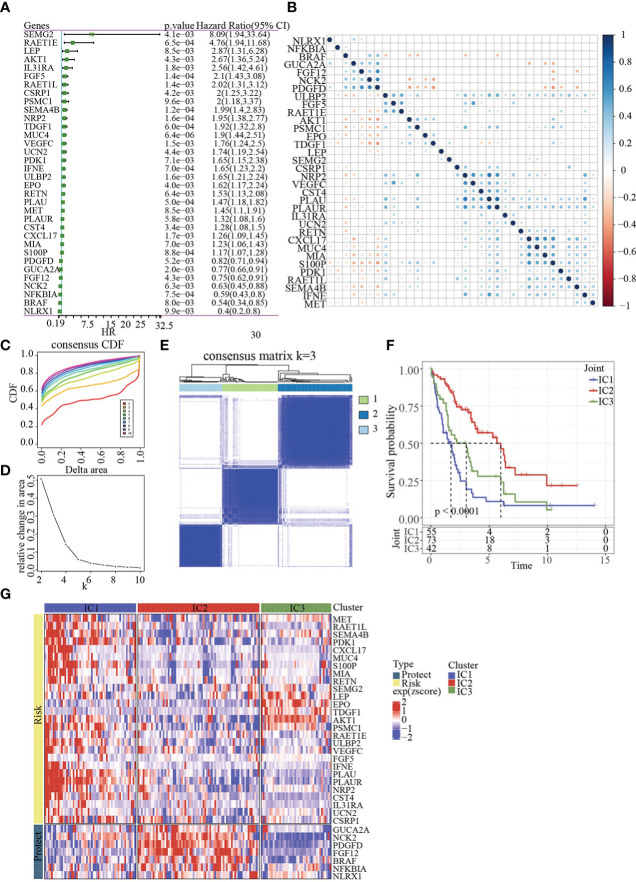
Identification of molecular subtypes. **(A)** Forest map of immune genes with significant prognosis analysed by univariate Cox regression. **(B)** Pearson correlation analysis of immune genes associated with prognosis. **(C)** Cumulative distribution function. **(D)** Delta area of Cumulative distribution function. **(E)** Clustering heatmap of samples in Joint data when k=3. **(F)** KM prognosis curve of 3 molecular subtypes. **(G)** Heatmap of immune genes associated with prognosis in 3 molecular subtypes.

The workflow was showed in **Figure 1**. The Limma package’s removeBatchEffect function was used to remove the batch effect of the three datasets, and the results showed no significant differences between the samples (**Figures 2A, B**). Univariate Cox survival regression analysis identified 34 immune genes associated with prognosis, including 27 risk genes and 7 protective genes (**Figure 3A**). Pearson correlation analysis showed that these genes were related to each other (**Figure 3B**).

Base on 34genes, the samples in Joint data were clustered with CDF and delta area (**Figures 3C, D**). When k=3, 3 clusters (IC1, IC2 and IC3) were found (**Figure 3E**). The survival of IC2 was better in Joint data, as shown by KM analysis (p<0.0001, **Figure 3F**). Heatmap analysis showed the expression of 34 genes in three molecular subtypes (**Figure 3G**).

### 3.2 Analysis of immune infiltration and immunotherapy

**Figure 4:**
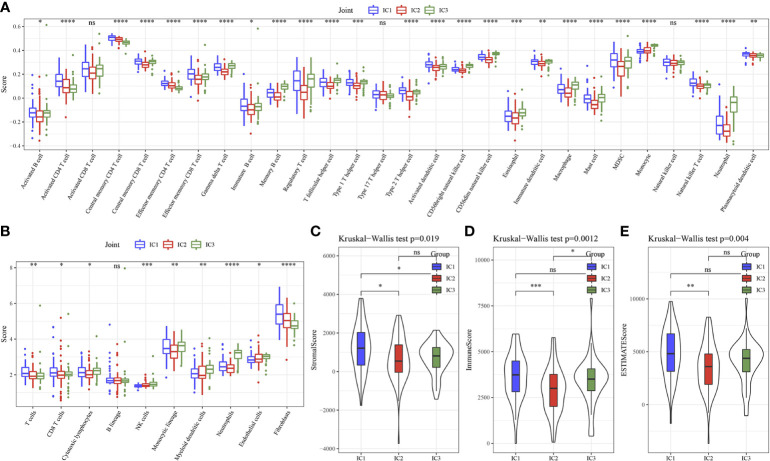
Immune characteristics of 3 molecular subtypes. **(A)** 28 immune cells scores differences of 3 molecular subtypes determined by ssGSEA. **(B)** 10 immune cells scores differences of 3 molecular subtypes determined by MCP-Counter. **(C)** StromalScore difference of 3 molecular subtypes determined by ESTIMATE. **(D)** ImmuneScore difference of 3 molecular subtypes determined by ESTIMATE. **(E)** ESTIMATEScore difference of 3 molecular subtypes determined by ESTIMATE. * p<0.05, ** p<0.01, *** p<0.001, **** p<0.0001, ns, no sense.

Here, 25 out of 28 immune cells had significantly difference using GSEA analysis among 3 clusters (**Figure 4A**). MCP-Counter analysis demonstrated that 9 immune cells had obviously differences among 3 clusters (**Figure 4B**). Then, higher score of ImmuneScore and ESTIMATEScore, StromalScore in IC1 was found using ESTIMATE analysis (**Figures 4C–E**).

**Figure 5:**
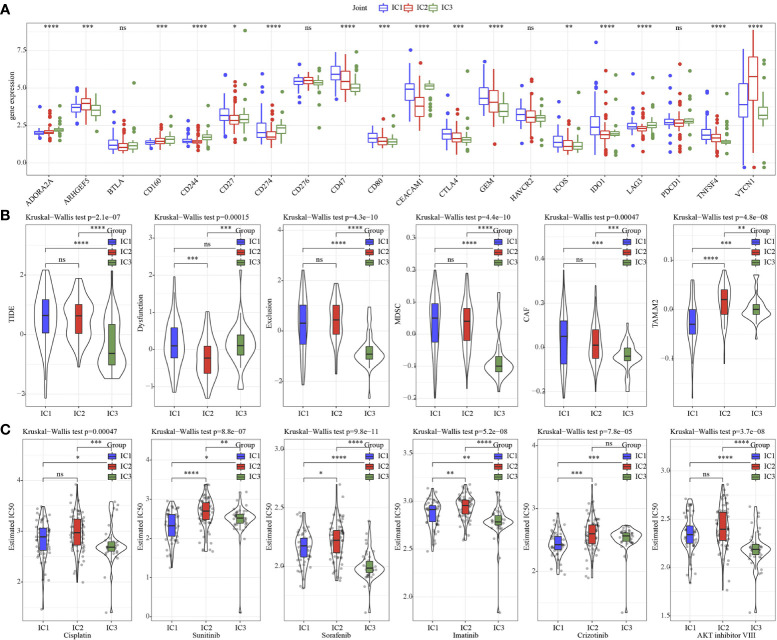
Immunotherapy analysis. **(A)** The expression levels of 20 immune checkpoint genes in 3 molecular subtypes. **(B)** TIDE analysis in 3 molecular subtypes. **(C)** The box plots of the estimated IC50 for Cisplatin, Sunitinib, Sorafenib, Imatinib, Crizotinib and AKT inhibitor VIII in 3 molecular subtypes. * p<0.05, ** p<0.01, *** p<0.001, **** p<0.0001, ns, no sense.

Next, the 20 immune check genes expressions were analyzed, and 16 immune checkpoint genes had obviously high expressions in IC1 that those in IC2 and IC3 (**Figure 5A**). TIDE, Exclusion, MDSC, CAF and TAM.M2 were lower in IC3 group, while Dysfunction was higher inIC3 group (**Figure 5B**), suggesting that IC3 group was more likely to benefit from immunotherapy. IC50 of Cisplatin, Sunitinib, Sorafenib, Imatinib, Crizotinib and AKT inhibitor VIII was lower in IC3, which suggested that IC3 was more sensitive to chemotherapeutic drug (**Figure 5C**).

### 3.3 Establishment of an immune related prognosis model for cholangiocarcinoma

**Figure 6:**
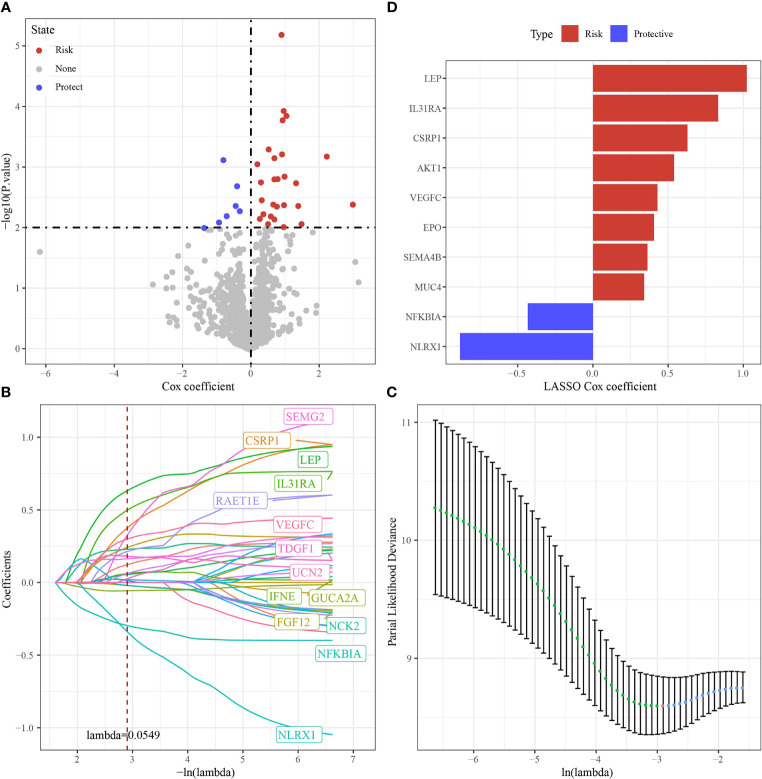
Identification of hub immune genes. **(A)** A total of promising immune genes candidates were identified through Lasso Cox regression. **(B)** The trajectory of each independent variable as lambda changes. **(C)** Confidence intervals under lambda. **(D)** Distribution of LASSO coefficients of the immune prognostic gene signature.

Based on 34 immune genes associated with prognosis (**Figure 6A**), LASSO Cox regression module was conducted to build a prognostic signature based on the expression matrix of the 10 genes. Here, we identified a 10-genes signature module according to the optimal λ value (**Figures 6B, C**). The distribution of Lasso coefficients of the immune prognostic gene signature was shown in **Figure 6D**. RiskScore of cholangiocarcinoma patients with 10 genes calculated followed the above formula: The RiskScore=0.429*VEGFC -0.434*NFKBIA-0.884*NLRX1+0.54*AKT1+0.629*CSRP1+0.406*EPO+0.833*IL31RA+1.023*LEP+0.341*MUC4+0.363*SEMA4B.

### 3.4 Validation of prognostic model

**Figure 7:**
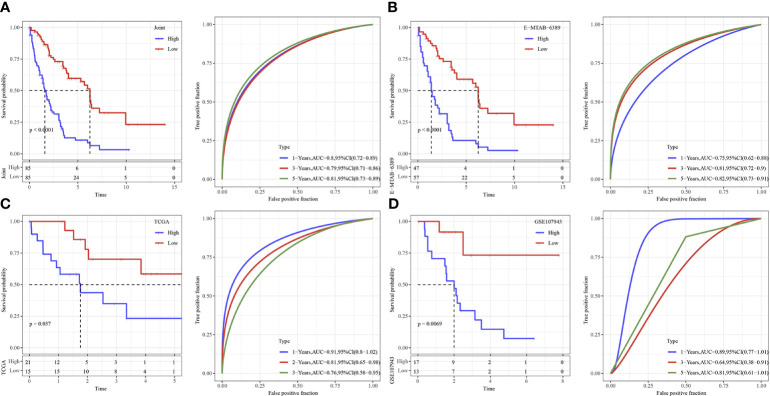
Validation of immune genes signature. **(A)** The KM curve and ROC analysis of RiskScore in Joint data. **(B)** The KM curve and ROC analysis of RiskScore in E−MTAB−6389 dataset. **(C)** The KM curve and ROC analysis of RiskScore in TCGA dataset. **(D)** The KM curve and ROC analysis of RiskScore in GSE107943 dataset.

The cutoff value taken here for high-risk and low-risk group classification in Joint data, TCGA-cholangiocarcinoma, GSE107943 and E-MTAB-6389 dataset was determined as the median value of the RiskScore. ROC and survival studies was conducted on the Joint data (**Figure 7A**), E-MTAB-6389 dataset (**Figure 7B**), TCGA-cholangiocarcinoma dataset (**Figure 7C**) and GSE107943 dataset (**Figure 7D**). From the current data, the model accuracy in prediction of 1‐, 3‐, and 5‐year survival rates in above datasets was higher, moreover, the area under the curve (AUC) exceeded 0.64. Overall survival was higher in low-risk group than high-risk group, as shown in the results of Kaplan-Meier survival analysis.

**Figure 8:**
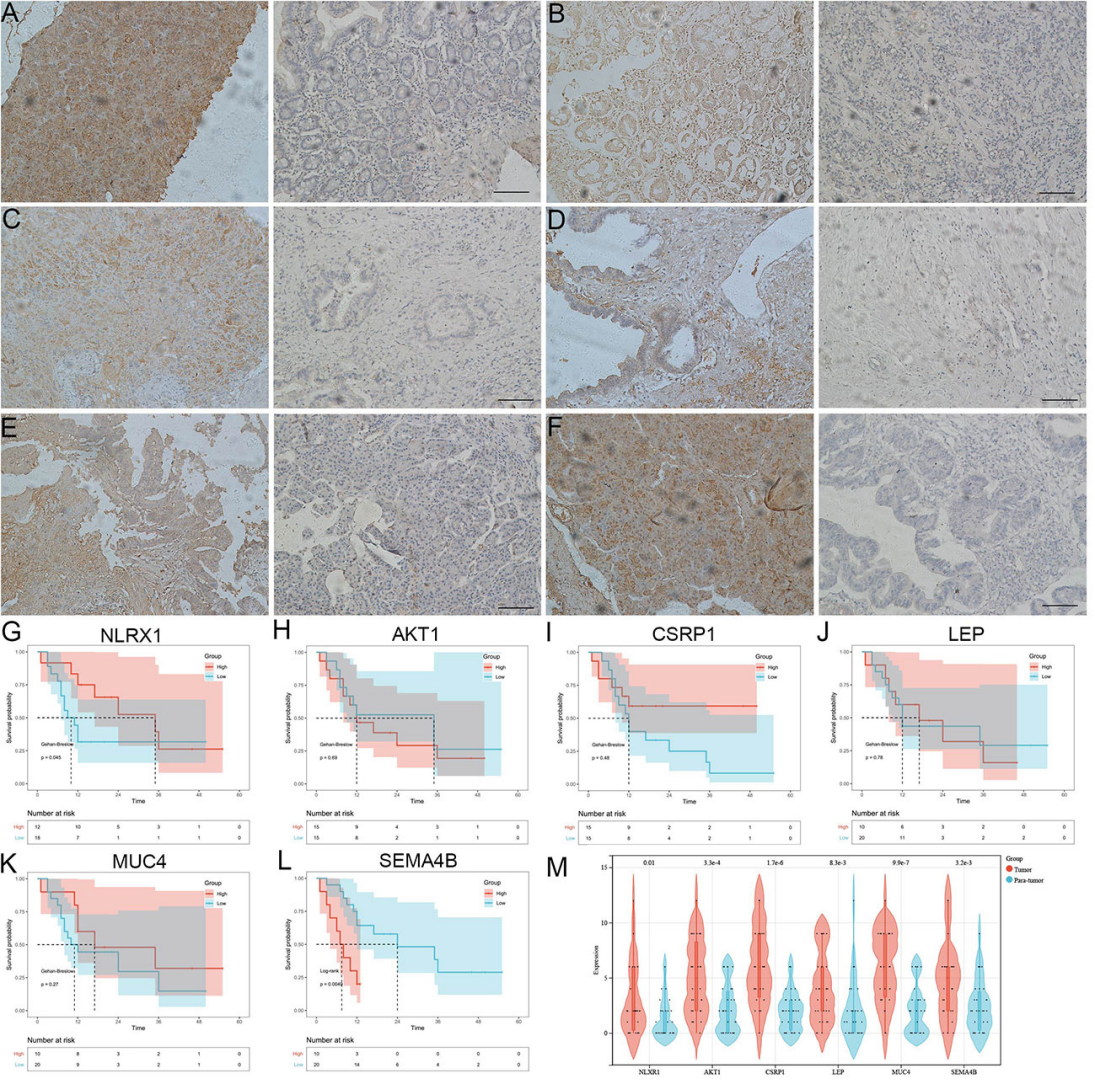
The analysis 6 genes using immunohistochemistry and KM survival curve. **(A-F)** the expression dysregulations of NLRX1, AKT1, CSRP1, LEP, MUC4 and SEMA4B in cancer tissues by immunohistochemistry. **(G-L)** Survival was better in patients with high NLRX1 expression and low SEMA4B expression compared with those with low NLRX1 expression and high SEMA4B expression. **(M)** 6 genes had difference expressions between cancer tissue and para-carcinoma.

In addition, we observed the expression dysregulations of NLRX1, AKT1, CSRP1, LEP, MUC4 and SEMA4B in cancer tissues by immunohistochemistry (**Figures 8A–F**). Survival was better in patients with high NLRX1 expression and low SEMA4B expression compared with those with low NLRX1 expression and high SEMA4B expression (**Figures 8G–L**). Moreover, those 6 genes also had difference expressions between tumor and para-tumor using online data analysis (**Figure 8M**).

### 3.5 Immune microenvironment and Functional enrichment analysis

**Figure 9:**
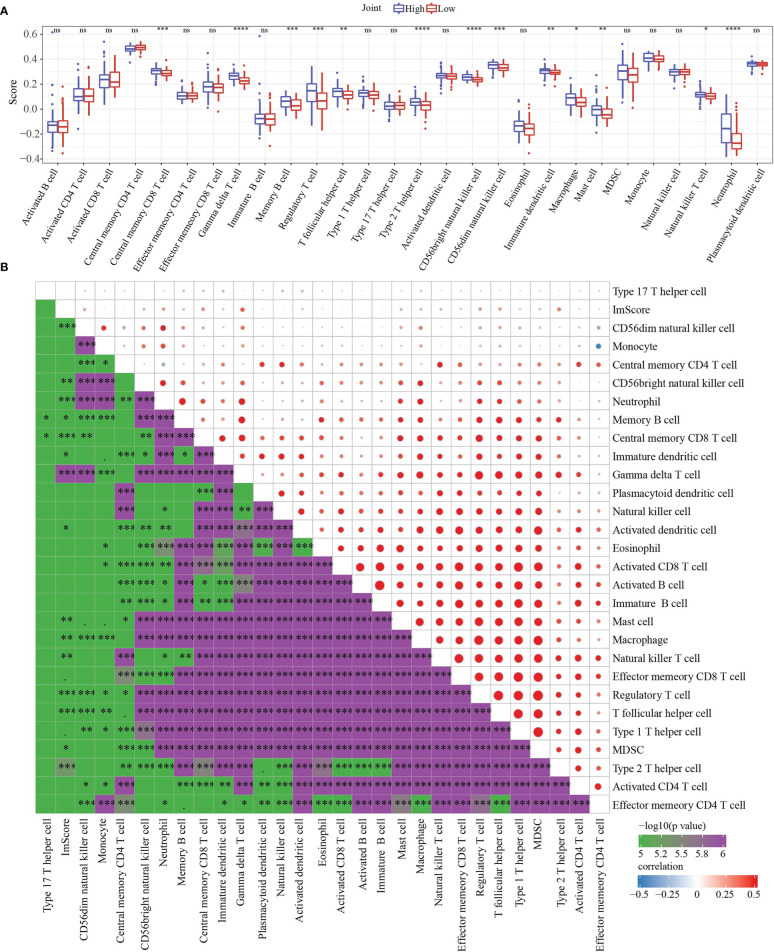
Immune characteristics and Functional enrichment analysis. **(A)** 28 immune cells scores differences between high group and low group determined by ssGSEA. **(B)** the correlation analysis between RiskScore and 28 immune cells scores. * p<0.05, ** p<0.01, *** p<0.001, **** p<0.0001, ns, no sense.

To further elucidate differences in the immune microenvironment of patients in the high- and low- group, we assessed immune cell infiltration in Joint data by using expression levels of genetic markers in 28 immune cells. The analysis demonstrated that in high group 13 immune cells score were higher (**Figure 9A**). RiskScore was positively correlated with 27 immune cells score after analyzing the relationship of 28 immune cells with RiskScore (**Figure 9B**).

**Figure 10:**
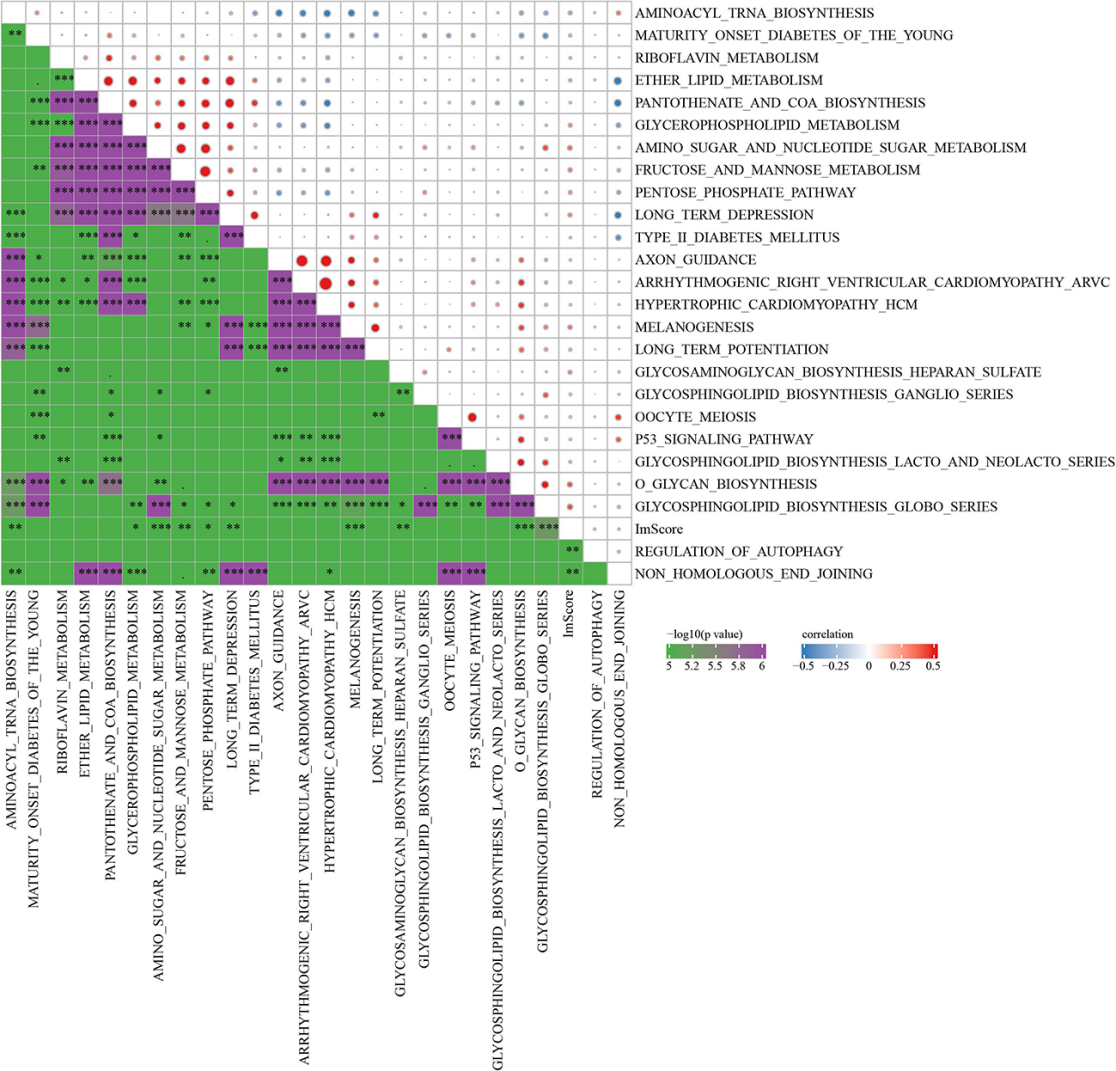
Correlation analysis between KEGG pathway and RiskScore with correlation greater than 0.2 in Joint data. * p<0.05, ** p<0.01, *** p<0.001.

The R software package GSVA was used to conduct single-sample GSEA analysis (ssGSEA) for examining the relationship between RiskScore and biological functions, and each sample’s score on different functions were counted. Further calculation of the relationship of RiskScore with these functions was executed, with the functions showing a correlation higher above 0.2 being selected (**Figure 10**). Those data showed that RiskScore was correlated immune related pathways.

### 3.6 Immunotherapy analysis

**Figure 11:**
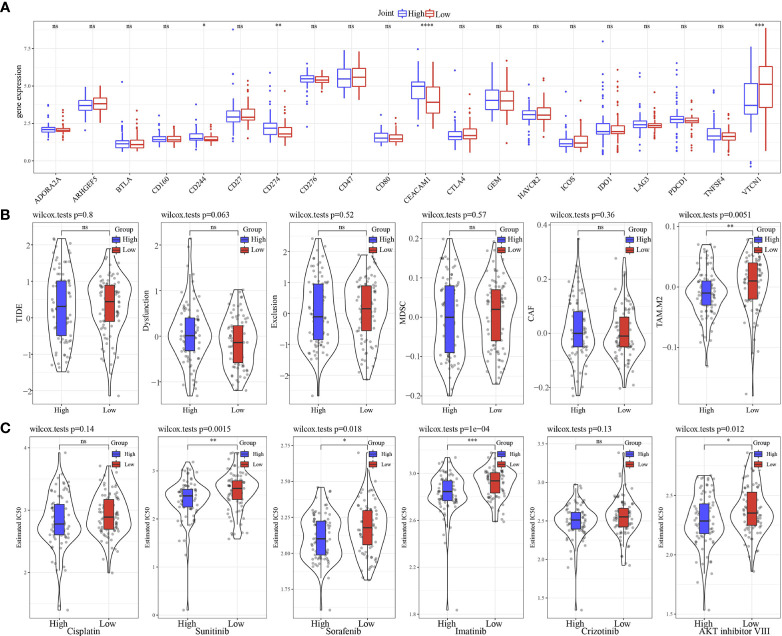
Immunotherapy analysis. **(A)** The expression levels of 20 immune checkpoint genes in high group and low group. **(B)** TIDE analysis in high group and low group. **(C)** The box plots of the estimated IC50 for Cisplatin, Sunitinib, Sorafenib, Imatinib, Crizotinib and AKT inhibitor VIII in high group and low group. * p<0.05, ** p<0.01, *** p<0.001, **** p<0.0001, ns, no sense.

We found that only 4 of 20 immune check genes expressions had difference expressions between high group and low group (**Figure 11A**). Only TAM.M2 was lower in high group (**Figure 11B**). IC50 of Sunitinib, Sorafenib, Imatinib, and AKT inhibitor VIII was lower in high group, indicating the potential of the model to be applied in sensitivity prediction of chemotherapeutic drug (**Figure 11C**). Those data showed that our model may sensitive to traditional medicine.

### 3.7 Performance examination of RiskScore in Crohn’s disease

**Figure 12:**
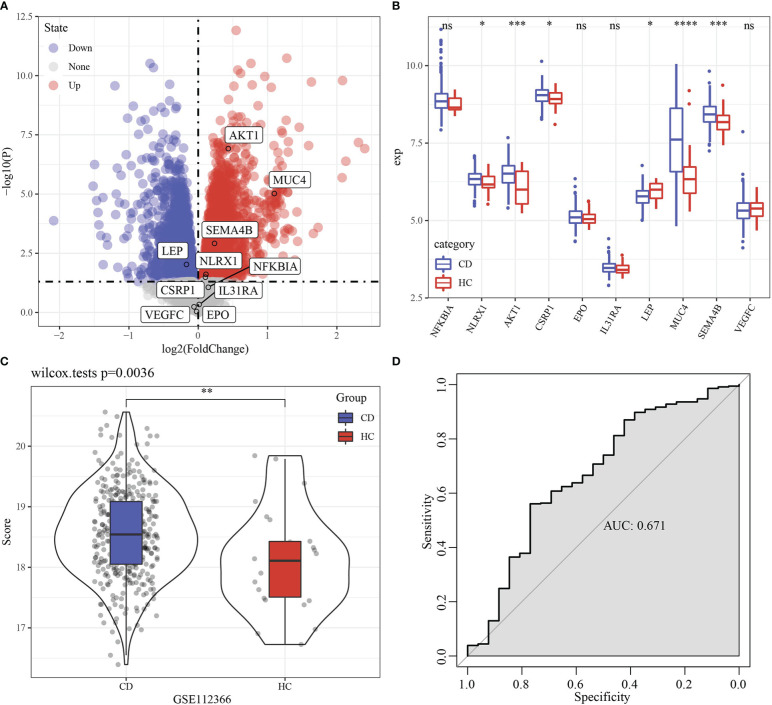
Performance examination of RiskScore in Crohn’s disease. **(A)** Volcano Plot of differentially expressed genes in GSE112366 dataset. **(B)** Boxplot of differentially expressed genes between Crohn’s disease and healthy samples in GSE112366 dataset. **(C)** RiskScore difference of between Crohn’s disease and healthy samples in GSE112366 dataset. **(D)** ROC curve of RiskScore in dataset GSE112366 dataset. * p<0.05, ** p<0.01, *** p<0.001, **** p<0.0001, ns, no sense.

Firstly, limma analysis was used to identify differentially expressed gene between 362 Crohn’s disease and 26 healthy samples, which came from GSE112366 dataset. We found that 6 of 10 model genes had differences (**Figures 12A, B**). Based on 10 genes model, the scores of Crohn’s disease were higher than that in healthy samples (**Figure 12C**). RiskScore was used to predict Crohn’s disease with an AUC value of 0.671 (**Figure 12D**).

## 4 Discussion

A total of 34 immune genes significantly affected cholangiocarcinoma prognosis, as shown by Univariate Cox regression analysis, and a complex correlation network of gene expression among the 34 genes was observed, indicating that the degree of immune infiltration was closely related to these genes. Therefore, through the 34 immune genes, cholangiocarcinoma was divided into 3 immune gene-related subtypes, and the results showed that in patient survival among the 3 subtypes statistically significant differences were observed. To explore the underlying mechanisms responsible for the survival differences among the three immune subtypes, immune infiltration analysis was performed. The results of immune infiltration analysis showed that the infiltration degree of antigen presenting B cells, dendritic cells, macrophages, tumor killing natural killer cells, CD8+T cells in IC1, IC3 subtype with relatively poor prognosis was significantly higher than that of IC2, which had a favorable prognosis. Analysis of tumor microenvironment demonstrated that the immune status was enhanced in IC1 and IC3. However, we further analyzed the high expression of LAG3 in IC1 and IC3, suggesting the existence of T cell depletion. NK cells could kill tumor cells through death receptor-mediated apoptosis and cytotoxicity mediated by granzyme. More importantly, NK cells can kill tumor cells in the cycle to prevent tumor metastasis (20). In the clearance of tumor cells CD8+T cells play an important role. Chronic inflammation and persistent antigenic stimulation of tumor can lead to depletion of CD8+T cells (21). Therefore, we hypothesized that T cell depletion might be the reason for a high immune infiltration and poor prognosis of IC1 and IC3. Emerging cancer vaccines and immunotherapy with immune checkpoint inhibitors are sensitive to cholangiocarcinoma (22).

Previously, a 6 immune-related genes signature predicts the survival outcome in advanced intrahepatic cholangiocarcinoma (23). For cholangiocarcinoma, previous study developed an 8-immune-related differentially expressed genes (8-IRDEGs) signature that showed a better prediction value (24). Here, mining, statistical analysis and collation of TCGA, GEO, EBI and IMPORT datasets identified 10 prognostically specific immune-related genes. Among all 10 prognostic specific immune-related genes, 4 genes (AKT1, EPO, MUC4, VEGFC) (25–29) were considered as important predictors of relapse-free or overall survival and were implicated in immune microenvironment-related pathogenesis of cholangiocarcinoma. Favorable prognosis of intrahepatic cholangiocarcinoma may be related to a dysregulated p-AKT1 expression (26). A progressive increase of EPO and EpoR mRNA can already be observed in cholangiocarcinoma (27). MUC4 is a novel prognostic factor of intrahepatic cholangiocarcinoma (30). VEGF-C mRNA transcription level showed a significant upward trend in cholangiocarcinoma cell lines treated with gemcitabine (31). Moreover, our model also had a sound performance in Crohn’s disease. Current bioinformatics analyses based on TCGA, GEO, EBI, and IMPORT cohorts have shown prognosis importance. Previously, association of the remaining six genes with cholangiocarcinoma prognosis and its role as novel markers for cholangiocarcinoma have not been found. These genes included NFKBIA, NLRX1, CSRP1, IL31RA, LEP, SEMA4B.

Though the prediction potential of immune scoring system for immunotherapy effect has been verified in this study to a certain extent, still, certain limitations of the study should be addressed. Firstly, the current research was dependent on TCGA and GEO (the mRNA expression data), that is to say there would be obvious ethnic specificity. Thus, its application to other ethnic populations requires further verification. Also, a small size of the study cohort was small asks for validation by with a larger sample number in immunotherapy cohort. Finally, study objects in the current study all derived from publicly available databases, statistically significant genes were introduced for developing the model, moreover, relationship of clinical immunotherapy effect and etiology required further verification. The clinical value of this model needs further experimental verification.

In conclusion, three immune gene subtypes of cholangiocarcinoma were identified in this study, and their differences in prognosis and immune cell infiltration were statistically significant. An immune gene model was constructed, with a prediction significance in the effect of immunotherapy for cholangiocarcinoma patients. This model could improve immunotherapy for patients with cholangiocarcinoma, and providing guidance in making clinical diagnosis, medication, prognosis related decision for cholangiocarcinoma patients with different immunophenotypes.

## Data availability statement

The datasets presented in this study can be found in online repositories. The names of the repository/repositories and accession number(s) can be found in the article/supplementary material.

## Author contributions

Z-jZ designed and wrote the paper; Y-pH, Z-tL, Y-xW, HZ, K-xH, and J-wT edited the work; YW, S-fH, and LX reviewed and revised the manuscript. All authors agree to be accountable for the content of the work. All authors contributed to the article and approved the submitted version.
